# Identification of novel B cell epitopes in Fiber-2 protein of duck adenovirus 3 and their application

**DOI:** 10.1186/s13568-023-01552-9

**Published:** 2023-06-22

**Authors:** Yun Lin, Wenyuan Zhang, Jing Xie, Weikang Wang, Quan Xie, Tuofan Li, Hongxia Shao, Aijian Qin, Zhimin Wan, Jianqiang Ye

**Affiliations:** 1 Key Laboratory of Jiangsu Preventive Veterinary Medicine, Key Laboratory for Avian Preventive Medicine, Ministry of Education, College of Veterinary Medicine, Yangzhou University, Yangzhou, 225009 Jiangsu China; 2 Jiangsu Co-innovation Centre for Prevention and Control of Important Animal Infectious Diseases and Zoonoses, Yangzhou, 225009 Jiangsu China; 3 Joint International Research Laboratory of Agriculture and Agri-Product Safety, the Ministry of Education of China, Yangzhou University, Yangzhou, 225009 Jiangsu China

**Keywords:** Duck adenovirus 3, Fiber-2 protein, Monoclonal antibodies, Epitope mapping, Sandwich ELISA

## Abstract

Duck adenovirus 3 (DAdV-3), a newly emerged duck adenovirus, has resulted in significant economic losses to the duck industry across China since 2014. However, little is known about the B cell epitopes in major antigen of DAdV-3 and the serological approach for detection of DAdV-3 is not available. In this study, four monoclonal antibodies (mAbs) specific to Fiber-2 protein of DAdV-3 were first generated and designated as 2G10, 3D9, 5E6, and 6B12. Indirect immunofluorescence assay (IFA) showed that all of the mAbs reacted with the Fiber-2. Moreover, mAbs 2G10, 5E6, and 6B12 demonstrated good activity with Fiber-2 in Western blot. Notably, the Fiber-2 could be immunoprecipitated efficiently by mAb 3D9. Epitope mapping revealed that mAbs 2G10, 3D9, 5E6, and 6B12 recognized 397-429aa, 463-481aa, 67-99aa, and 1-66aa of Fiber-2, respectively. Besides, a novel sandwich ELISA for efficient detection of DAdV-3 was developed based on mAb 3D9 and horseradish peroxidase (HRP) conjugated mAb 3D9. The sandwich ELISA only reacted with DAdV-3 but not with other duck-associated viruses. The limit of detection of the ELISA was 6.25 × 10^3^ TCID_50_/mL. Overall, the mAbs generated laid the foundation for elucidating the critical role of Fiber-2 in mediating infection and pathogenesis, and the sandwich ELISA approach established here provided efficient and rapid serological diagnostic tool for DAdV-3.

## Key points

1. Four mAbs against Fiber-2 of DAdV-3 were generated.
2. Four novel B cell epitopes in Fiber-2 of DAdV-3 were identified.
3. An efficient sandwich ELISA for detection of DAdV-3 was developed.

## Introduction

According to the International Committee on Taxonomy of Viruses (ICTV) (https://talk.ictvonline.org/taxonomy/), the family Adenoviridae is divided into five genera: *Atadenovirus*, *Aviadenovirus*, *Ichtadenovirus*, *Mastadenovirus*, and *Siadenovirus*. So far, two duck-derived adenoviruses are clearly classified by the ICTV: DAdV-1 in the genus *Atadenovirus* (Cha et al. 2013) and DAdV-2 in the genus *Aviadenovirus* (Marek et al. 2014). DAdV-1, also known as egg drop syndrome virus (EDSV), caused markedly decrease in egg production and quality in ducks (Das and Pradhan 1992). In 1982, DAdV-2 infection in Muscovy ducks was reported in France for the first time. Infected ducks lost weight, and some were lame (Bouquet et al. 1982). In 2014, the infection of DAdV-3, as a novel disease, broke out in many duck farms across southern China. In the last few years, DAdV-3 was widespread and some mutants with higher pathogenicity were circulated in Fujian, Zhejiang, Anhui and Guangdong of China (Shi et al. 2022; Yin et al. 2022). In 2019, DAdV-4 was isolated from some dead Jinding ducks charactered by salpingitis in Guangdong of China (Huang et al. 2020).

The disease of DAdV-3, characterized as swelling and hemorrhagic liver and kidneys, caused substantial economic losses to the duck industry (Shi et al. 2019). DAdV-3, similar to other adenoviruses, is a medium-sized, non-enveloped icosahedral virion with linear, non-segmented, double-stranded DNA genome (Davison et al. 2003). The genome encodes four major structural proteins, including Hexon, Penton, and two Fibers (Fiber-1 and Fiber-2). Among them, two Fibers play vital roles in mediating the infection and inducing immune responses (Yin et al. 2019). Notably, as an efficient protective antigen, the Fiber-2 protein could induce significant humoral antibody to offer full protection against DAdV-3 infection (Yin et al. 2019). However, the B cell epitopes in this dominant antigen have not been identified and there is no any licensed serological method specific for clinical detection of DAdV-3. In this study, four novel mAbs specific to the Fiber-2 protein of DAdV-3 were generated, and their epitopes were identified. Moreover, an efficient mAb-based sandwich ELISA for specific detection of DAdV-3 was established.

## Materials and methods

### Viruses, cells and plasmids

DAdV-1, DAdV-3, serotype 4 fowl adenovirus 4 (FAdV-4), H9N2 avian influenza virus, Tembusu virus (TMUV), and goose astrovirus type 1 (GAstV-1) were maintained in our laboratory. Duck hepatitis virus type 1 (DHV-1), duck hepatitis virus type 3 (DHV-3), duck reovirus (DRV), and avian paramyxovirus type 4 (APMV-4) were provided kindly by Prof. Zongyan Chen (Shanghai Veterinary Research Institute). LMH cells from ATCC were cultured in DMEM/F12 (Gibco, NY, USA) supplemented with 10% FBS (Lonsera, Shanghai, China) at 37 °C with 5% CO2. Plasmids pcDNA3.1 and pCold-1 were stored in our laboratory. pcDNA3.1-Fiber-2 carrying with the Fiber-2 of DAdV-3 was generated and stored in our laboratory.

### Expression and purification of recombinant Fiber-2

The recombinant plasmid pCold-1-Fiber-2 was constructed by full-length *Fiber-2* gene (GenBank accession number: ON995379) and plasmid pCold-1 digested with *EcoR* I and *Xho* I restriction enzyme. The corresponding primers were listed in Table 1. The recombinant plasmid pCold-1-Fiber-2, confirmed by sequencing, was transformed into *E. coli* BL21 (DE3) cells. A single positive *E. coli* BL21 colony was cultured overnight and the culture was inoculated into 1 L LB medium with a ratio of 1:100 at 37 °C. When the OD value reached 0.6, isopropyl β-D-thiogalactoside (IPTG) was added with a final concentration of 1mM for overnight induction at 16 °C. The *E. coli* BL21 cells transformed with pCold-1-Fiber-2 were collected, resuspended in binding buffer (10 mM Na_2_HPO_4_, 10 mM NaH_2_PO_4_, 500 mM NaCl, 20 mM imidazole, pH 7.4), ultrasonicated, and then centrifuged at 13,000 g at 4 °C for 20 min. The Ni-NTA agarose column filled with the supernatant was incubated on a four-dimensional rotating mixer at 4 °C for 2 h, then washed with binding buffer, and eluted with elution buffer (10 mM Na_2_HPO_4_, 10 mM NaH_2_PO_4_, 500 mM NaCl, 600 mM imidazole, pH 7.4).

**Table 1:** Primers for construction of plasmids

Name	Direction	Sequence(5’–3’)
pCold-F2-F	Forward	CCGCTCGAGATGAAACGGACCAACAGATC
pCold-F2-R	Reverse	CCGGAATTCCTAATTAACATTTGATGGGTTG
pc-F2-F	Forward	AGCTTGGTACCGAGCATGAAACGGACCAACAGATC
pc-F2-R	Reverse	ATATCTGCAGAATTCCTAATTAACATTTGATGGGTTG
pc-F2-1-66aa-R	Reverse	ATATCTGCAGAATTCTTACGGGTCGGATGTTCTTACGT
pc-F2-1-99aa-R	Reverse	ATATCTGCAGAATTCTTATAGAGGCCCGTTTGGGTCGA
pc-F2-1-132aa-R	Reverse	ATATCTGCAGAATTCTTAGATAGGGCCACGTGGATCTG
pc-F2-1-165aa-R	Reverse	ATATCTGCAGAATTCTTATAGTCGTACACCGAGAGTCA
pc-F2-1-198aa-R	Reverse	ATATCTGCAGAATTCTTAACTAACGAGTACTGTTAGGC
pc-F2-1-231aa-R	Reverse	ATATCTGCAGAATTCTTATAACTCTTTCTCTCCTTGAG
pc-F2-1-264aa-R	Reverse	ATATCTGCAGAATTCTTATAGGGTATTGTCAGTGACGG
pc-F2-1-297aa-R	Reverse	ATATCTGCAGAATTCTTACGTAACAGACCCTGCTCCGC
pc-F2-1-330aa-R	Reverse	ATATCTGCAGAATTCTTATACAGCATGTGATGCATCCT
pc-F2-1-363aa-R	Reverse	ATATCTGCAGAATTCTTAGTTGTTGGTACAGTGGGAAA
pc-F2-1-396aa-R	Reverse	ATATCTGCAGAATTCTTACAGAATAAAGTCATGTTCTG
pc-F2-1-429aa-R	Reverse	ATATCTGCAGAATTCTTATACTTGGGCTATGGAGAATC
pc-F2-1-462aa-R	Reverse	ATATCTGCAGAATTCTTAATCACCAGTTGTGCCTTGTT
pc-linear-F	Forward	GAATTCTGCAGATATCCAGCACAGTG
pc-linear-R	Reverse	GCTCGGTACCAAGCTTAAGTTTAAACG

### Generation of mAbs against Fiber-2 protein of DAdV-3

Purified His-Fiber-2 fusion protein mixed with Freund’s complete adjuvant (Sigma-Aldrich, Missoula, MO, USA) was injected into 6-week-old BALB/c mice for the first immunization. For subsequent immunizations, the Freund’s complete adjuvant was instead by Freund’s incomplete adjuvant (Sigma-Aldrich). There is a two-week interval between the injection of Fiber-2 protein. During the interval, the antibody titers of the sera from the immunized mice were detected. When the antibody titers of the sera were high enough, the Fiber-2 protein without adjuvant was injected for the last time. After three days, the prepared SP2/0 cells were fused with the spleen cells of immunized mice by PEG1500 (Roche, Mannheim, Germany) as previously described (Wang et al. 2018). On the tenth day after the cell fusion, the supernatants of the hybridoma cells were detected using LMH cells infected with DAdV-3 through IFA. The positive hybridoma clones were subcloned, and the isotypes of the generated mAbs were determined with a mouse mAb isotyping kit (Thermo Scientific, Massachusetts, USA) according to the manufacturer’s instructions.

### Indirect immunofluorescence assay

LMH cells infected with DAdV-3 or transfected with pcDNA3.1-Fiber-2 and its serial truncations were fixed with the fixative solution (the prechilled acetone and ethanol with the ratio of 3:2) for 5 min. Then, the prepared corresponding mAbs diluted were incubated with the fixed LMH cells for 45 min at 37 °C. After three washes with PBS, the LMH cells were incubated with FITC-conjugated goat anti-mouse IgG (Sigma-Aldrich, USA) for another 45 min. After three washes with PBS, the cells were observed using an inverted fluorescence microscope.

### Western blot

LMH cells infected with DAdV-3 or transfected with pcDNA3.1-Fiber-2 were washed and lysed with RIPA buffer (CWbio, Beijing, China) containing protease and phosphatase inhibitors (CST, MA, USA). The lysates mixed with loading buffer were boiled for 10 min and centrifuged for 3 min. After SDS-PAGE, the denatured proteins were transferred onto nitrocellulose membrane (GE Healthcare Life sciences, Freiburg, Germany). After blocked with 5% skim milk in PBST for 2 h at room temperature (RT), the membrane was incubated with the corresponding mAbs diluted with 5% skim milk in PBST for 2 h at RT. After three washes with PBST, the membrane was incubated with HRP-conjugated goat anti-mouse IgG diluted with 5% skim milk in PBST for 1 h. After another three washes, the membrane was developed with chemiluminescent reagents and imaged with an automatic imaging system (Tanon 5200).

### Immunoprecipitation

The Thermo Scientific™ Pierce™ Crosslink Magnetic IP/Co-IP Kit was used for immunoprecipitation according to the manufacturer’s instructions. First, the purified mAb was coupled to the protein A/G magnetic beads, and they were covalently crosslinked with disuccinimidyl suberate (DSS). The mAb-crosslinked beads were washed to remove mAb that was not covalently bound. After incubated with the lysate of LMH cells transfected with pcDNA3.1-Fiber-2 or infected with DAdV-3 for 1 h, the prepared beads were washed to remove non-bound material and eluted in a low-pH elution buffer that dissociated the bound Fiber-2 protein from the antibody-crosslinked beads. The eluted low-pH sample, neutralized by neutralization buffer, was applied to Western blot analysis.

### B cell epitope mapping

The recombinant plasmid pcDNA3.1-Fiber-2 and its serial truncations were constructed based on the linearized plasmid pcDNA3.1 and the corresponding fragments using ClonExpress II One Step Cloning kit (Vazyme, Nanjing, China). The corresponding primers were listed in Table 1. After sequenced to confirm the correctness, the plasmids with different truncations of Fiber-2 were transfected into LMH cells respectively. Then the transfected cells were fixed with fixative solution and incubated with corresponding mAbs and secondary antibody. The epitopes were finally identified by IFA.

### Alignment of epitope sequences in Fiber-2 protein

To analyze the conservation of the four epitopes identified, the sequence alignment was performed using Clustal W program based on the software DNAStar (DNAStar Inc., Madison, WI, USA). The Fiber-2 protein sequences of DAdV-3, DAdV-4 and Fiber protein sequences of DAdV-1, DAdV-2 were downloaded from GenBank (http://www.ncbi.nlm.nih.gov). All of the downloaded sequences were put into DNAStar together for alignment.

### mAb-based sandwich ELISA

To develop sandwich ELISA for detection of DAdV-3, mAb 3D9 was purified and HRP-conjugated as previously described (Wang et al. 2018). In the sandwich ELISA, the purified mAb 3D9 diluted in 0.1 M carbonate buffer with the final concentration of 1.25 µg/mL was coated into a 96-well ELISA plate (100 µL/well) at 4 °C overnight. The following morning, the 96-well ELISA plate was blocked with 360 µL of 5% skim milk in PBST for 2 h at 37 °C. After the blocking and washes, the virus supernatants or samples diluted were added into the 96-well ELISA plate for 1 h at 37 °C and the 96-well ELISA plate was washed with PBST for three times. Then, the HRP-conjugated mAb 3D9, diluted by PBST with a final concentration of 0.625 µg/mL, was added into the 96-well ELISA plate for 30 min at 37 °C. After washed with PBST for three times, the 96-well ELISA plate was added with 100µL TMB single-component substrate solution (Solarbio) per well and reacted for 15 min at 37 °C. Finally, 50 µL of 2 M H_2_SO_4_ per well was used to stop the chromogenic reaction and an ELISA reader was used to measure the absorbance values at 450 nm.

## Results

### Expression and purification of recombinant Fiber-2 protein of DAdV-3

**Fig. 1:**
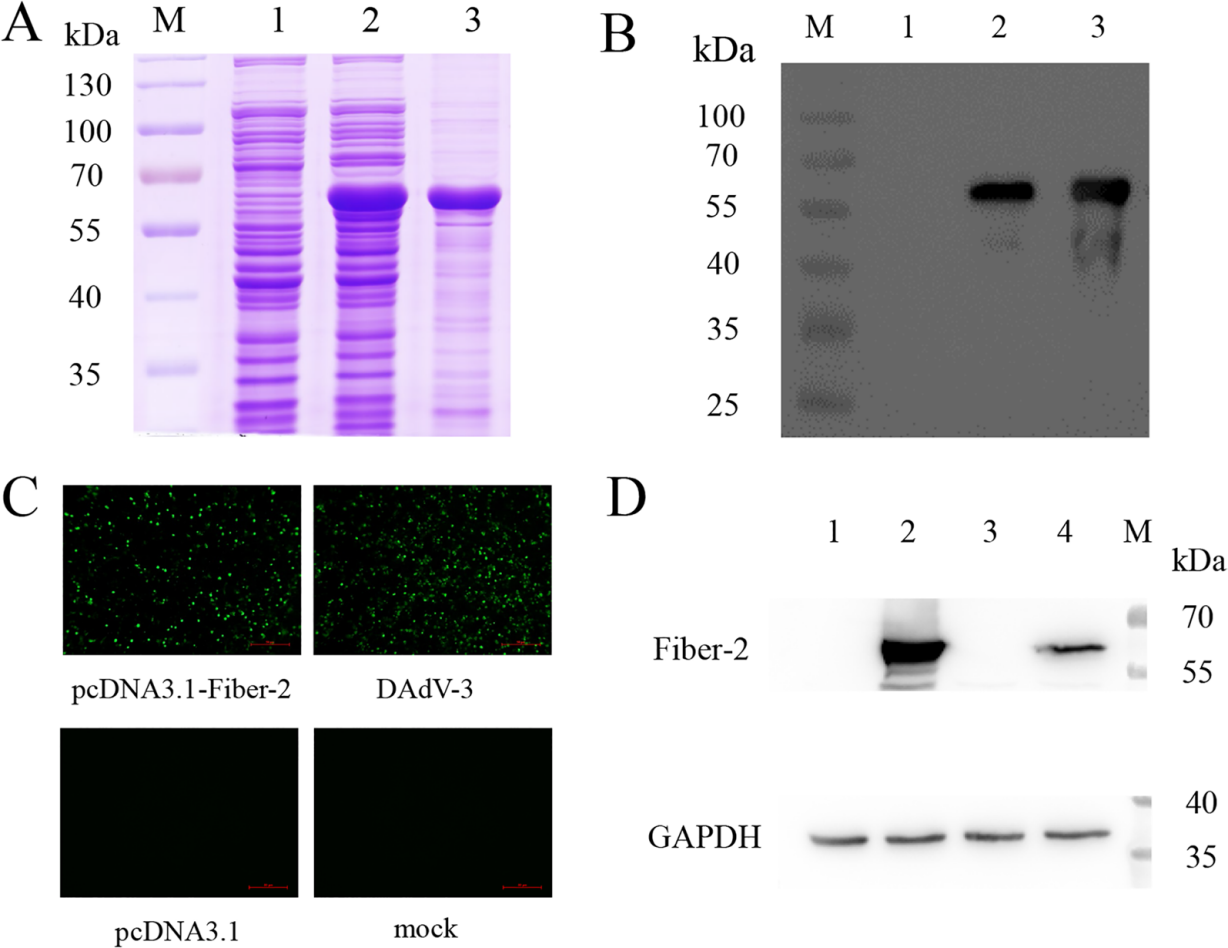
The expression of the recombinant Fiber-2 and its immunogenicity. The expressed and purified recombinant Fiber-2 was identified by SDS-PAGE (**A**) and Western blot (**B**) with mAb against His-Tag as primary antibody. M, PageRuler™ Prestained Protein Ladder; Lane 1: bacterial lysate of *E. coli* BL21 transfected with pCold-1; Lane 2: bacterial lysate of *E. coli* BL21 transfected with pCold-1-Fiber-2; Lane 3: His-Fiber-2 fusion protein purified from bacterial lysate of *E. coli* BL21 transfected with pCold-1-Fiber-2. The polyclonal antibody was identified with transfected or infected LMH cells by IFA (**C**) and Western blot (**D**). Lane 1: LMH cells transfected with pcDNA3.1; Lane 2: LMH cells transfected with pcDNA3.1-Fiber-2; Lane 3: LMH cells mock infected; Lane 4: LMH cells infected with DAdV-3.

In order to obtain the Fiber-2 protein, the recombinant plasmid pCold-1-Fiber-2 carrying the Fiber-2 of DAdV-3 was first constructed and then transformed into the *E. coli* BL21 cells. The transformed BL21 cells were then induced by IPTG, and the expressed recombinant His-Fiber-2 fusion protein was purified using Ni-NTA agarose column. As showed in Fig. 1A and B, after the expression and purification, the recombinant Fiber-2 protein displayed an apparent molecular weight of 55 kDa measured by SDS-PAGE and Western blot, which was in agreement with the expected size of the expressed product. These data demonstrated that the prokaryotic recombinant Fiber-2 protein of DAdV-3 was successfully expressed and purified.

### Generation and characterization of four mAbs specific to Fiber-2 protein

**Fig. 2:**
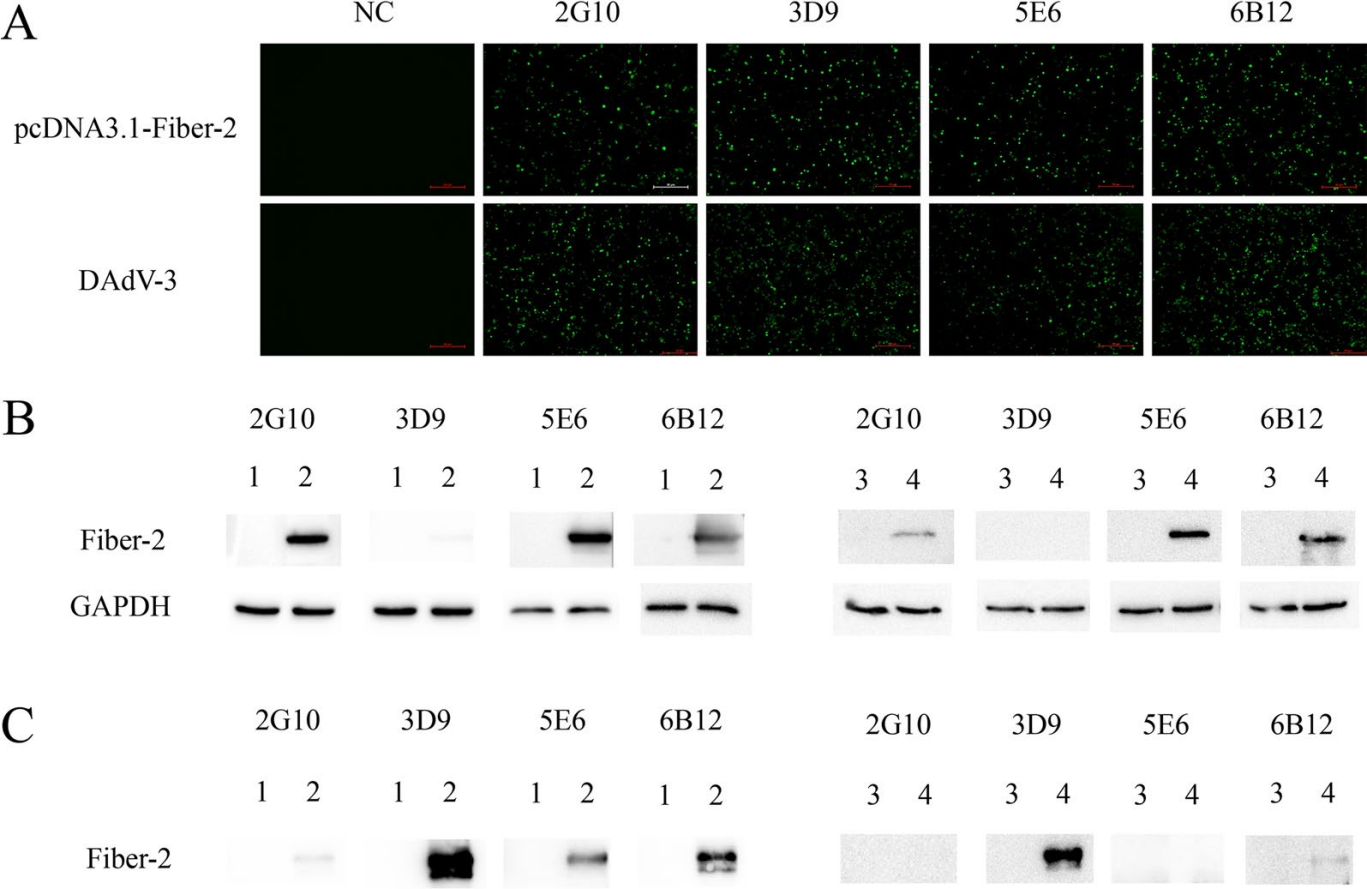
The characteristics of mAbs against Fiber-2. The reaction profiles of mAbs were analyzed with transfected and infected LMH cells by IFA (**A**), Western blot (**B**) and immunoprecipitation (**C**). Lane 1: LMH cells transfected with pcDNA3.1; Lane 2: LMH cells transfected with pcDNA3.1-Fiber-2; Lane 3: LMH cells mock infected; Lane 4: LMH cells infected with DAdV-3.

To generate mAbs against Fiber-2 protein of DAdV-3, BALB/c mice were immunized with the purified His-Fiber-2 fusion protein. LMH cells infected with DAdV-3 or transfected with pcDNA3.1-Fiber-2 were used as antigens for the detection of sera from immunized mice. As shown in Fig. 1C and D, the sera of the immunized mice had good reactivity with DAdV-3 and its Fiber-2 protein in IFA and Western blot. After fusion and subcloning, four mAbs, designated as 2G10, 3D9, 5E6, and 6B12, showed strong positive reaction. As described in Fig. 2A, the four mAbs could react not only with LMH cells infected with DAdV-3 but also with LMH cells transfected with pcDNA3.1-Fiber-2 by IFA. Moreover, mAbs 2G10, 5E6, and 6B12 could recognize the linear epitopes of Fiber-2 protein in both infected and transfected LMH cells by Western blot as described in Fig. 2B. Besides, the Fiber-2 protein could be immunoprecipitated efficiently by the purified mAb 3D9 (Fig. 2C). The subtypes of the four mAbs 2G10, 3D9, 5E6, and 6B12 were identified as IgG1, IgG2a, IgG1, and IgG2a respectively using a mouse mAb isotyping kit (Table 2). All these data not only demonstrated the good immunogenicity of the expressed Fiber-2 protein of DAdV-3, but also provided mAbs for developing efficient detection approaches for DAdV-3.

**Table 2:** Identification of the isotype of the mAbs

Name	2G10	3D9	5E6	6B12
Isotype	IgG1	IgG2a	IgG1	IgG2a

### Identification of novel B cell epitopes in Fiber-2 protein

**Fig. 3:**
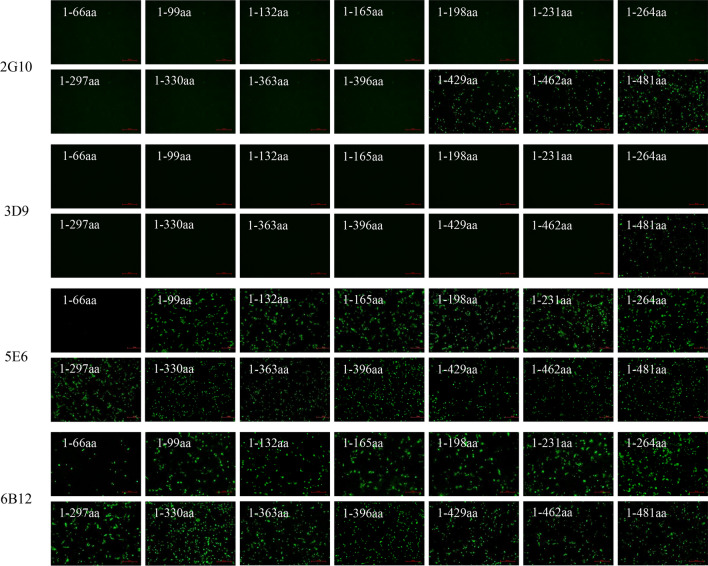
Mapping of the B cell epitopes in Fiber-2 protein. The LMH cells transfected with the different Fiber-2 truncations were respectively analyzed using mAb 2G10, 3D9, 5E6, and 6B12 by IFA.

**Fig. 4:**
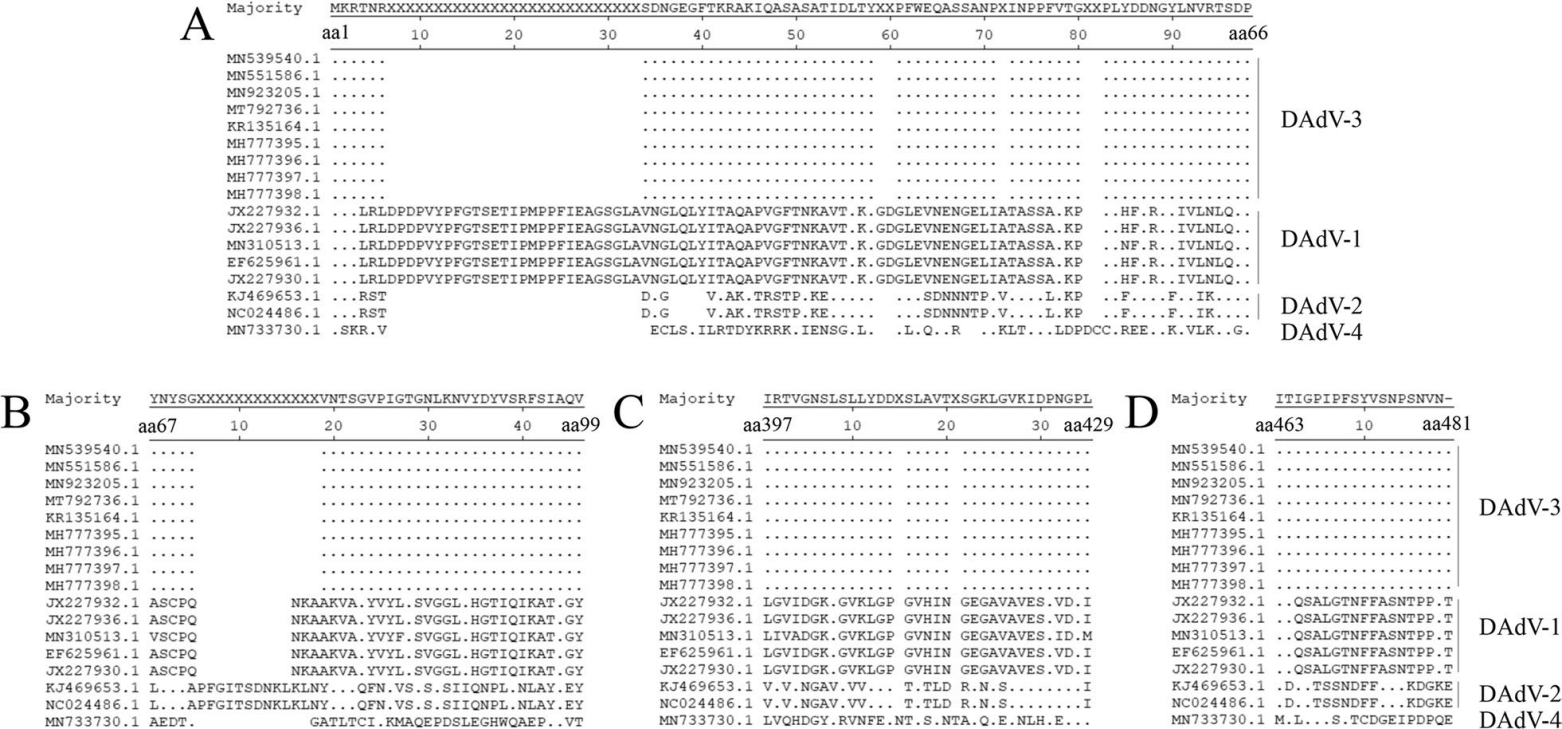
Analysis of the epitopes recognized by the mAbs. Sequence comparison analysis of the four epitopes recognized by 6B12 (**A**), 5E6 (**B**), 2G10 (**C**), and 3D9 (**D**) was performed with different DAdV stains

To determine the B cell epitopes recognized by the generated mAbs specific to Fiber-2, fourteen Fiber-2 truncations with deletions at C terminus, designated as 1-66aa, 1-99aa, 1-132aa, 1-165aa, 1-198aa, 1-231aa, 1-264aa, 1-297aa, 1-330aa, 1-363aa, 1-396aa, 1-429aa, 1-462aa and 1-481aa, were constructed with the primers listed in Table 1. Notably, all these truncations of Fiber-2 could be efficiently expressed and recognized by mAb 6B12 as shown in Fig. 3. According to the reaction profile of the four mAbs as described in Fig. 3, the B cell epitopes recognized by mAbs 2G10, 3D9, 5E6, and 6B12 were located in 397-429aa, 463-481aa, 67-99aa, and 1-66aa of Fiber-2 protein, respectively. To evaluate whether these identified epitopes in Fiber-2 were conserved, the sequences of Fiber-2 protein in DAdV-3, DAdV-4 and Fiber protein in DAdV-1, DAdV-2 were aligned. As showed in Fig. 4A, B, C and D, the four epitopes were completely consistent among DAdV-3 strains, whereas they were significantly different from the other types of DAdVs tested here. These data demonstrated that the four epitopes identified in Fiber-2 protein were highly conserved among DAdV-3, highlighting that these mAbs could be used for specific diagnostics of DAdV-3.

### Application of mAb 3D9 in serological approach for detection of DAdV-3

**Fig. 5:**
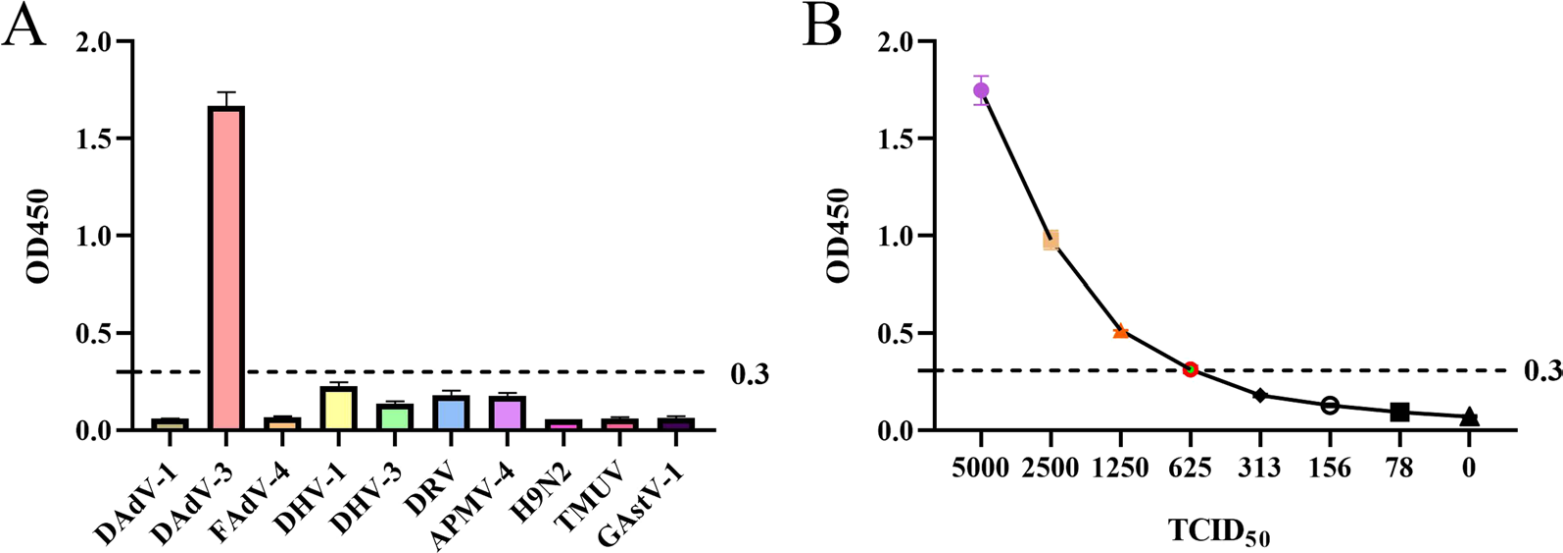
Development of an efficient mAb 3D9-based sandwich ELISA.** A** Specificity of mAb-based sandwich ELISA was analyzed using different viruses, including DAdV-1, FAdV-4, DHV-1, DHV-3, DRV, APMV-4, H9N2 AIV, TMUV and GAstV-1. The error bars represent the results of three experimental repeats. **B** Sensitivity of mAb-based sandwich ELISA was analyzed by the diluted DAdV-3 with the indicated viral titers. The error bars represent the results of three experimental repeats

To investigate whether the mAbs could be used in the development of serological approach for detection of DAdV-3, the purified and HRP-conjugated mAb 3D9 was applied to develop a sandwich ELISA. The cut-off value of the sandwich ELISA was defined as the mean value plus threefold standard deviation of the other duck-derived virus supernatants. Therefore, the cut-off value was finally determined as 0.3. The specificity analysis showed that the sandwich ELISA could specifically detect DAdV-3 with high OD450 value of 1.67, and the OD450 values of other duck-derived viruses, including DAdV-1, FAdV-4, DHV-1, DHV-3, DRV, APMV-4, H9N2 AIV, TMUV, and GAstV-1, were very low and under the cut-off value as described in Fig. 5A. The sensitivity analysis demonstrated that the limit of detection of the ELISA was 6.25 × 10^3^ TCID_50_/mL as shown in Fig. 5B. All these data demonstrated that the developed sandwich ELISA with good specificity and sensitivity could be applied for the detection of DAdV-3.

## Discussion

DAdV-3, an emerging duck adenovirus, resulted in large economic losses to duck industry in China (Shi et al. 2019). Up to now, the extensive and in-depth researches about the pathogen, pathogenicity, epidemiology, rapid diagnosis, and vaccine were carried out to prevent and control DAdV-3 infection (Chen et al. 2019; Shi et al. 2019; Wan et al. 2018; Yin et al. 2019; Zhang et al. 2016). Although indirect ELISA or qPCR methods to detect antibody or DNA of DAdV-3 have been established (Chen et al. 2019; Wan et al. 2018), the mAb-based serological method for detection of antigen for DAdV-3 was not available so far. Notably, as a conservative surface protein of the viral particle, the Fiber-2 protein is not only an efficient target for the establish of the detection method but also an immunogen to induce the production of neutralizing antibodies (Yin et al. 2019). However, little is known about the B cell epitopes in the Fiber-2 protein and the molecular mechanism for the infection and pathogenesis of DAdV-3 also needs to be further elucidated.

To obtain valuable mAbs against Fiber-2 for clinical application and elucidating its function in viral infection and pathogenesis, the Fiber-2 protein was expressed in *E.Coli* and purified successfully with an approximate size of 55 kDa, which was consistent with the data published (Chen et al. 2019). The purified Fiber-2 protein was used as an antigen to immunize the mice, and then four mAbs 2G10, 3D9, 5E6, and 6B12 specific to Fiber-2 protein were efficiently generated. Notably, all these mAbs developed here could efficiently react with Fiber-2 protein in LMH cells either infected with DAdV-3 or transfected with pcDNA3.1-Fiber-2 through IFA. Among these mAbs, mAb 3D9 did not recognize the linear epitope in Western blot in comparison with other three mAbs, but mAb 3D9 could efficiently immunoprecipitate the Fiber-2 protein expressed in LMH cells either infected with DAdV-3 or transfected with pcDNA3.1-Fiber-2 as described in Fig. 2C. Further epitope mapping revealed that mAbs 2G10, 3D9, 5E6, and 6B12 recognized different epitopes (397-429aa, 463-481aa, 67-99aa, and 1-66aa, respectively) in the Fiber-2. Moreover, these epitopes reacted with the four mAbs were highly conserved across DAdV-3 strains, but significantly variant in other duck adenoviruses tested, highlighting the specificity of these mAbs for DAdV-3 and their application in diagnostics. To establish an efficient sandwich ELISA for detection of DAdV-3, mAb 3D9 and HRP-conjugated mAb 3D9 was selected as the capture antibody and the detection antibody, respectively. In our initial study, we also tried other mAbs to use as the capture antibody or the detection antibody in the sandwich ELISA. However, other mAbs did not work well as mAb 3D9 did in the sandwich ELISA, possibly due to different epitopes recognized by these mAbs. mAb 3D9 recognized the conformational epitope and had high affinity with the Fiber-2 in immunoprecipitation, which might contribute to the high efficacy of mAb 3D9 in the sandwich ELISA. The sandwich ELISA developed here only reacted with DAdV-3, not with other viruses tested, and had high sensitivity with the LOD of 6.25 × 10^3^ TCID_50_/mL, highlighting the potential application in clinical detection of DAdV-3.

In summary, this is the first demonstration of the generation of four mAbs against Fiber-2 of DAdV-3 and identification of novel B cell epitopes in the Fiber-2 protein. The identified epitopes recognized by these mAbs are highly conserved in DAdV-3, but significantly variant in other duck adenoviruses tested. The established mAb 3D9-based sandwich ELISA also shows high specificity and sensitivity for the detection of DAdV-3. The generated mAbs, defined B cell epitopes, and established ELISA not only provide novel insights into the DAdV-3, but also pave the way for further elucidating the roles of Fiber-2 in the infection and pathogenesis, and developing efficient diagnostic tools for specific detection of DAdV-3.

## Data Availability

The datasets used and analyzed during the current study are available from the corresponding author on reasonable request.
